# The Value of Post-Graduate Instruction

**Published:** 1893-07

**Authors:** G. Lenox Curtis

**Affiliations:** New York


### THE VALUE OF POST-GRADUATE INSTRUCTION.[1]

BY G. LENOX CURTIS, M.D., D.D.S., NEW YORK.

MANY of you will doubtless recall the fierce attack upon the dental colleges made by the Philadelphia *Medical Times* some years ago, in which the editor (Professor H. C. Wood) stigmatized their degree "D.D.S." as the "badge of a partial culture." You will recall also how the late Dr. J. W. White, who took up the cudgels for the dental schools, showed from Professor Wood's own testimony that they were at least doing what they claimed to do,—grounding their students in the principles of dental practice,—which, if the same witness was to be believed, was a good deal more than could be hoped for from the medical colleges of the day, notwithstanding

---

[1] Read before the Central Dental Association of Northern New Jersey, March 17, 1893.

the *Times* held them up as the only proper educators for practitioners of dentistry as of any other specialty in medicine.

The sense in which the *Times* used the phrase "partial culture" was that the dental college could not, *per se*, fit a student for the practice of dentistry, because it did not and could not give him the proper medical knowledge. It is not necessary now to enter into the merits of that contention, but there is a broader sense in which the D.D.S., when granted even after the most thorough course of instruction enforced in any dental college, is the "badge of a partial culture." But it is no more so than is the M.D. of the callow medical graduate, the diploma of the unadmitted law-school fledgling. Each of these bears testimony to the fact that its holder has passed the required curriculum of study. The young dental graduate, for instance, has been taught the general principles of practice, but his time has mostly been occupied in acquiring knowledge, so that when graduated he is merely ready to learn how to apply that knowledge. He is, however, extremely ready, his training having made him apt in the absorption and application of methods. As Dr. Kirk puts it, "The most that has been absorbed when the diploma has been won is a knowledge of the more important principles, a familiarity with typical methods, a limited facility in technique, and, what is equally important, if not more so, the establishment of a habit and method of study giving the ability to readily acquire further knowledge and practically apply it as occasion arises."

The bare statement of the acquirements of the recent graduate testifies his need for further study. He is ready to "begin" practice, of course, but who will say he is prepared to cope with the complicated conditions which, common enough in their occurrence, yet test the resources of even the skilled practitioner? Of theoretical knowledge there is no lack. So far as this phase of his qualifications is concerned, the recent graduate can almost surely pass a better examination than his more skilled brother of twenty years' active practice; better fourfold than he himself can when twenty years of active work have made him a shining light in his profession. What he stands most in need of now is the fertility of resource, the keen discernment, the accurate diagnosis, the trained judgment, and, most of all, the manual dexterity, which twenty years of active practice will give. How shall he conquer these, so that patients at the outset of his career shall have as useful service as they will receive at his hands twenty years after? It is most of all in the face of this lack that his D.D.S. is the "badge of a partial culture."

Dr. S. B. Palmer, in a paper published in the INTERNATIONAL DENTAL JOURNAL for February, states the weakness of college instruction when he says, "There is not sufficient time spent with students in manual training to enable them to meet the requirements of prosthetic dentistry; so when they graduate they have made one or two practical dentures and from twelve to fifty fillings in all."

How is this to be remedied? There seems but one answer,—by the post-graduate school. Extension of the term of studentship has done much to correct evils formerly existent in our scheme of education. There is now no lack of theoretical knowledge of the science of dentistry in the average graduate. His greatest weakness is in the direction of manual dexterity, which, after all, stands very near the first essential to the practical dentist. A fourth year in college, of exclusively clinical and practical work under the guidance of skilled instructors, would meet the difficulty to a large degree; but even then the student would not have sufficient opportunity to decide certainly what particular line of work he would wish to follow, and as no man can be an expert in all things, a year or two of private work would be required to show him what he really needed, when the post-graduate school would afford the means to supply his wants.

Then, again, there is that large class of men who have entered upon practice without the advantage of a college training. Many of these would be eager to avail themselves of the opportunities for practical instruction to be found alone in a properly-conducted post-graduate school or practitioner's course.

If dentistry hopes to maintain the dignity which it now claims, it must lay out a course of clinical study covering the higher grades of work, to be taught by men having the requisite knowledge and the ability to impart it. By this means the average ability of the profession at large would be sensibly increased. Not every man who is perfectly competent to deliver a lecture before a class of raw students is fit to demonstrate the fine arts of delicate manipulation before a body of graduates and practitioners. These do not sit in open-mouthed wonder as at revelations of an occult science. They are prepared to question whatever is not clear, and they test with more or less keenness the practicality of what is offered. Their instructor must needs know his subject thoroughly, must not merely be "up" in its present development, but must be familiar with the steps by which that development has been reached, even the errors which experience has demonstrated; in a word, he must be a real expert in the particular branch he essays to teach. Dentistry does not lack for such men. What it does lack is the opportunity and the means to avail itself of their knowledge, their skill, and their willingness to give of their store to the general stock of knowledge, which the general establishment of post-graduate schools would make available. Indeed, in view of the importance of the subjects involved, it may be a question whether attendance upon post-graduate instruction should not be made obligatory upon all graduates.

Men whose good fortune it has been to take a course or courses in post-graduate work are loud in its praises. It is well to read in the journals descriptions of practical methods of procedure; well to hear them discussed in society meetings. It gives one food for thought, broadens the mind, perhaps suggests new ideas. But the moral effect is as nothing compared with seeing every step of the operation, and then under the eye of the instructor doing it one's self. It gives one confidence in himself, a state which always inspires the confidence of his patients.

In these days, for instance, no dentist can be accounted an all-round, finished workman who does not possess a thorough knowledge of crown- and bridge-work. Yet it is safe to say not four per cent. of practitioners have this knowledge. Many more think they have it; but they judge from a biased stand-point,—their own work, not that of experts. To acquire this knowledge requires special training, without which the proper practice of this important branch of our work is impossible to the great mass of dentists. Its importance will be the better recognized when we reflect that it is the agency which is rescuing prosthetic dentistry from the low estate into which the introduction of vulcanite plunged it. Were it for no other reason than to assist in this rehabilitation in the esteem of all men of a once-honored department of dental practice, the knowledge of the principles and procedures of crown- and bridge-work should be universal. Since the introduction of this beautiful work we hear less and less of the one-time shibboleth, "I do no mechanical work," by which the sheep of the profession sought to distinguish themselves from its goats.

Few men fresh from college are able to construct a bridge properly; fewer still—and this applies also to the mass of practitioners—can do any real surgery in the mouth. The reason why is suggested by Dr. Palmer's remark before quoted. Most of the difficult work, such as surgery, bridge, and continuous gum, in the colleges is done by the professors or the demonstrators; so that a practical knowledge of more than the simplest operations is too seldom obtained. In a properly-conducted post-graduate school a vastly different rule prevails. It should be manned by competent instructors, experts in each branch such as I have previously described, who would be there to teach, not to practise; to show those sitting under him how to do and then supervise their efforts to do. That is the kind of instruction which is now mostly required to elevate the standard of dentistry. Rightly carried out it will place the schools upon the highest level which can be demanded of them.

To sum up its advantages, the post-graduate school brings the man who seeks it for instruction into close contact with greater men, suppresses his egotism, broadens his intellect, draws forth his noblest qualities, and inspires his highest ambitions; fosters his inventive faculty and instills into him a reverence for science; in a word, it places within the reach of every man who will learn a practical knowledge of the highest development of dental procedures, and by so much advances the true interests of the profession and of the race.

I have said that it is a question whether post-graduate work should not be made obligatory upon all graduates. I am persuaded that the experiment of a post-graduate school, founded upon the lines herein indicated and conducted with an eye single to the advancement of those in attendance, would, by its results, so react upon the moral sense of the profession as to make the post-graduate course, or its equivalent, a necessary preliminary to entrance upon practice.

